# Use of Epoprostenol in the Treatment of Pulmonary Arterial Hypertension

**DOI:** 10.7759/cureus.18191

**Published:** 2021-09-22

**Authors:** Asma Mohammadi, Wanessa F Matos, Cesar Intriago, Keval Thakkar, Nasrin Jahan, Heeya Shah, Rifath I Nishu, Sima Marzban

**Affiliations:** 1 Division of Research & Academic Affairs, Larkin Community Hospital, South Miami, USA

**Keywords:** prostacyclin, prostanoids, pah, pulmonary hypertension, epoprostenol

## Abstract

Pulmonary Hypertension (PH) is defined as a disorder in which the mean Pulmonary Arterial Pressure (mPAP) is greater than 20 mmHg at rest. Pulmonary Arterial Hypertension (PAH) is considered when mPAP is > 20 mmHg and pulmonary vascular resistance (PVR) is ⩾ 3 WU. PAH is a chronic progressive disease resulting in right heart failure and premature death. It is postulated to be due to an inactivating mutation of a gene named bone morphogenetic protein receptor type 2 (BMPR2), whose predominant function is halting vascular proliferation. It has a lamentable prognosis if not rapidly diagnosed and adequately treated. Treatment of PAH has evolved in the past few decades since many related pathways and potential therapeutic targets have been explored. Parenteral prostanoids are the most effective therapeutic options for PAH. Epoprostenol is a synthetic analog of prostacyclin and a potent vasodilator that was Food and Drug Administration (FDA)-approved in December 1995 for intravenous use to treat PAH. It has also been used to treat different PAH subtypes, including connective tissue-related PAH like lupus and systemic sclerosis, congenital heart disease, and drug-induced PAH. It is effective in reducing mortality rates and improving survival rates. Although the use of Epoprostenol for PAH is challenging, it has been one of the most successful therapies used. In this manuscript, we review the pathophysiology of PAH and the risk stratification tool. We also discuss the mechanism of action of PAH-targeted therapies while focusing on the role of epoprostenol that has been investigated in many clinical trials. Finally, we discuss two ongoing clinical trials which highlight some potential therapeutic options.

## Introduction and background

Pulmonary Hypertension (PH) is defined as a disorder in which the mean Pulmonary Arterial Pressure (mPAP) is greater than 20 mmHg at rest [1]. During exercise, 25 mmHg is taken into account for the clinical diagnosis of PH. Pulmonary arterial hypertension (PAH) is considered when mPAP is > 20 mmHg and pulmonary vascular resistance (PVR) is ⩾ 3 WU. Patients with pulmonary arterial pressures falling between 21 and 24 have demonstrated poor outcomes and are, therefore, considered at high risk. PAH is a debilitating disease that leads to pathological changes to the pulmonary arteries, involving vascular remodeling of the vessels, ultimately leading to right ventricular (RV) failure and death [2].

In the 20th century, PAH was considered to affect people aged 65 years and above. Currently, PAH has been identified to affect any age group but is common in the young and elderly. Registry to Evaluate Early and Long-term PAH (REVEAL), a multicenter observational study placed in the United States, estimated the incidence and prevalence of PAH in adults to be 2.3 and 12.4 cases per million respectively in 2011 [3]. The national registries and healthcare systems reported an incidence and prevalence of 5.8 and 47.6 - 54.7 per million worldwide in the last five years, respectively [4]. Although PAH was traditionally considered to affect young women, it is now known that PAH affects both genders considerably [5].

PAH involves proliferative changes in the lumen of the pulmonary arterial vessels. Pathophysiology mainly involves three pathways: endothelin (ET), nitric oxide (NO), and prostacyclin (PGI2) [6]. It is due to the imbalance between the vasoconstrictor and vasodilator components of the body [7]. In addition, it is often considered genetically associated due to the inactivating mutation of a gene named Bone morphogenetic protein receptor type II (BMPR2), which usually functions to stop vascular proliferation [7]. Therapies for PAH include pulmonary vasodilators like (epoprostenol, treprostinil, iloprost), guanylate cyclase (GSC) stimulators (Riociguat), and endothelin receptor antagonists (bosentan, macitentan, ambrisentan). If medications fail to control PAH, some surgical options include atrial septostomy and lung transplantation [8].

The World Health Organization (WHO) has proposed five groups based on the patient presentation, laboratory findings, treatment, and hemodynamic characteristics [1,9]. This segregation sought to standardize the treatment protocol and clinical approach.

1. Group I includes PAH due to idiopathic, heritable, drug-induced, and toxin-induced causes or is associated with the following: connective tissue diseases, portal hypertension, congenital heart disease, schistosomiasis, pulmonary veno-occlusive disease/or pulmonary capillary hemangiomatosis, and persistent pulmonary hypertension of the newborn (PPHN).

2. Group II includes PH due to left heart disease.

3. Group III includes PH due to lung disease and/or hypoxia.

4. Group IV includes PH due to pulmonary artery obstruction.

5. Group V includes PH due to unclear and/or multifactorial causes.

Risk assessment plays an essential role in delivering care and managing patients with PAH. The Registry to Evaluate Early and Long-term PAH Disease Management (REVEAL) has validated an effective score system to predict mortality and prognosis to provide appropriate treatment. It was updated in 2019 and is shown in Table 1 [10,11]. After calculating the variables, it is possible to find the risk score: 0 to six (low risk), seven to eight (intermediate risk), and > 9 (high risk) [12].

**Table 1:** REVEAL 2.0 (PAH Risk Score) WHO: World Health Organization, NYHA: New York Heart Association, BNP: B-type Natriuretic Peptide, NT Pro-BNP: N-terminal pro-B-type Natriuretic Peptide. Adapted from the original by Benza et al., [12].

Risk Stratification Groups	PAH Risk Score
WHO Group I (PAH) Subgroup - Associated – Connective tissue disease	1
WHO Group I (PAH) Subgroup- Associated – Portopulmonary hypertension	3
WHO Group I (PAH) Subgroup - Familial PAH	2
WHO Group I (PAH) Subgroup - Other	0
Males age >60 years old	2
Estimated glomerular filtration rate (eGFR) <60mL/min/1.73m² or renal failure	1
NYHA/WHO Functional Class I	-1
NYHA/WHO Functional Class II	0
NYHA/WHO Functional Class III	1
NYHA/WHO Functional Class IV	2
Systolic blood pressure <110 mmHg	1
Heart rate >96 bpm	1
All-Cause Hospitalizations ≤ 6 months	1
6-minute walk test >440 meters	-2
6-minute walk test 320 - 440 meters	-1
6-minute walk test 320 - 165 meters	0
6-minute walk test <165 meters	1
BNP <50 pg/mL	-2
BNP 50 - <200 pg/mL	0
BNP 200 - <800 pg/mL	1
BNP >800 pg/mL	2
NT pro-BNP <300 pg/mL	-2
NT pro-BNP 300 - <1100 pg/mL	0
NT pro-BNP >1100 pg/mL	2
Pericardial effusion seen on Echocardiogram	1
Pulmonary function test - % predicted diffusing capacity of the lungs for carbon monoxide (DLCO) <40	1
Mean right arterial pressure >20 mm Hg within one Year	1
Right heart catheterization - Pulmonary vascular resistance < 5 Wood units	-1
	Sum of the above score +6 = PAH risk score

According to the WHO Symposium guidelines, treatment of WHO group one PAH patients targets the nitric oxide, endothelin, and prostaglandin pathways [13]. Epoprostenol, a synthetic prostacyclin, improves symptoms, exercise capacity, and hemodynamics. It has been shown to reduce mortality in high-risk patients and benefits patients with PAH even as monotherapy [14]. Intravenous epoprostenol is the drug that has surfaced in the market and is becoming a success for treating PAH [15]. Supportive therapy includes physical activity, contraceptive usage, supervised rehabilitation, prevention of infection, anticoagulants use, oxygen, and diuretics, especially for right ventricular failure and fluid overload [16].

The first oral therapy approved for PAH was Bosentan, an Endothelin ET_A_ /ET_B _receptor antagonist. Pathophysiology of PAH involves a significant role of vasodilator pathways, as mentioned earlier. Therefore, it was necessary to further work on developing medications that affected these pathways. [13] The treatment for PAH patients has updated in the past decades, expanding in complexity and evidence for efficacy. Psychosocial support should be encouraged combined with the use of medication. Offering genetic counseling is a part of the treatment process for patients with sporadic or familial PAH or pulmonary veno-occlusive disease/pulmonary capillary haemangiomatosis (PVOD/PCH) [9].

The US Food & Drug Administration (FDA) has approved about 14 drugs [17]. These agents target three of the pathways involved in the pathological mechanism of PAH: endothelin, nitric oxide (NO), and prostacyclin (PGI2). The primary purpose of PAH therapy is to delay the progression of the disease. A double or triple combination of drugs to target multiple pathways concomitantly is an essential strategy for reaching this objective [18].

The FDA approved epoprostenol in December 1995 for intravenous (IV) use to treat PAH. Inhaled epoprostenol solutions also have been effectively used. In 2000, the FDA approved epoprostenol to treat pulmonary hypertension in scleroderma patients [19-21]. A new formulation approved by the FDA in 2010 is used to treat PAH associated with scleroderma, lupus, congenital heart disease, diet-pill associated, and stimulated associate PH [21]. Chen et al. conducted a systematic review in 2009, including 20 Randomized Control Trials. They evaluated the efficacy and cost-effectiveness of Epoprostenol, Iloprost, Bosentan, Sitaxentan, and Sildenafil for the treatment of PAH within their authorized indications. They found all these five drugs to be more effective than supportive treatment alone [22]. Nevertheless, combination therapy should be considered in New York Heart Association (NYHA) classes II, III, or IV based on the efficacy and evidence in small studies and retrospective studies and registries [23].

American College of Chest Physicians (CHEST) guidelines 2019 suggested combining World Health Organization Functional Class (WHO FC), exercise capacity, echocardiographic, laboratory, and hemodynamic variables to evaluate the severity of PAH and enlighten therapeutic decisions. Patients with PAH WHO FC III, rapid disease progression, or poor prognosis, if willing or able to manage parenteral prostaglandins, are candidates for continuous IV epoprostenol, which showed six-min walk distance (6MWD) improvement [17]. Furthermore, a meta-analysis for total mortality of the three randomized control trials with epoprostenol has established a risk reduction for mortality of about 70% [9].

Experts have recommended avoiding inhaled epoprostenol for non-intubated patients using a heated high-flow nasal cannula or venti mask. For intubated patients, as an ultimate indication, epoprostenol can be started at 0.01 - 0.05 mcg/kg/min and increased gradually. The ventilator filter should be changed every two hours due to glycine buffer diluent and the risk of rebound pulmonary hypertension due to ventilator clogging [24].

## Review

Pharmacodynamics

The pharmacological effects of epoprostenol are due to pulmonary and systemic arterial vasodilation. The effects on platelet aggregation are directly opposite to thromboxane A2 [25].

The inhibitory effect of epoprostenol on platelet aggregation is mediated by the stimulation of adenylate cyclase and the subsequent increase of cyclic adenosine monophosphate-3 ', 5' (cAMP) in platelets, leading to a rise in the intracellular levels of cyclic AMP. Sequential stimulation of adenylate cyclase followed by activation of phosphodiesterase has been described in human platelets [26].

Elevated levels of cAMP regulate intracellular calcium concentrations by eliminating it, which ultimately inhibits platelet aggregation due to the reduction of cytoplasmic calcium. Platelet shape variation depends on the degree of aggregation and the release reaction. The hemodynamic effects of Epoprostenol are caused by the increase of cyclic AMP in vascular smooth muscle and subsequent vasodilation. The hemodynamic effects of epoprostenol include reduced pulmonary vascular resistance, increased cardiac index, and increased oxygen delivery. However, hypotension is also a hemodynamic effect [27].

In humans, epoprostenol-induced hemodynamic changes (e.g., elevated heart rate, facial flushing) returned to baseline within 10 minutes after 60-minute infusions of 1 to 16 ng/kg/min were stopped. According to animal and in vitro studies, this pharmacodynamic activity is consistent with a limited in vivo half-life and rapid clearance in humans [28].

Pharmacokinetics 

Epoprostenol hydrolyzes quickly at neutral pH. Animal studies with epoprostenol show the clearance to be 93 mL/kg/min with a small amount distribution (357 mL/kg). It has a relatively short half-life of 2.7 minutes. The metabolization occurs primarily to two metabolites, 6-keto-PGF1α and 6,15-diketo-13,14-dihydro-PGF1α, due to spontaneous degradation and enzymatical formation, respectively. It is essential to point out that there is insufficient assay specificity and sensitivity to thoroughly assess Epoprostenol's pharmacokinetics. It is crucial to know the half-life of Epoprostenol (in vitro) is approximately six minutes. On the other hand, excretion is mostly via urine (84%) and feces (4%) [21,28].

Drug interactions 

More recently, literature and clinical practice have emerged, offering guidance. About 1,332 drugs can interact with epoprostenol. Practically, most of the adverse effects of Epoprostenol are based on the lowering of blood pressure. Some of the drugs that have the effect of lowering pressure are listed below [28,29]. 

Quinagolide, prostacyclin analogs, phosphodiesterase 5 inhibitors, pentoxifylline, acetazolamide, obinutuzumab, nitroprusside, nicorandil, nicergoline, naftopidil, molsidomine, lormetazepam, herbs (hypotensive properties), digoxin, diazoxide, brimonidine, bromperidol, antipsychotic agents [second generation (atypical)], alfuzosin [29,29].

Likewise, some medications have had adverse effects, especially agents with antiplatelet properties (e.g., P2Y12 inhibitors, NSAIDs, SSRIs, etc.). Additionally, anticoagulants and thrombolytic agents have specific adverse effects. These adverse effects are associated with increasing or decreasing the effects of anticoagulation [28,29].

Decrease

17-alpha-hydroxyprogesterone, allylestrenol, altrenogest, chlormadinone, chlorotrianisene, cyproterone, demegestone, desogestrel, dienogest, diethylstilbestrol, drospirenone, dydrogesterone, equol, estradiol, estradiol benzoate, estradiol acetate, estradiol cypionate, estradiol valerate, estriol, conjugated estrogens, esterified estrogens, estrone, ethinyl estradiol, ethynodiol, ethynodiol diacetate, etonogestrel, gestodene, gestrinone, gestonorone, hydroxyprogesterone caproate, levonorgestrel, lynestrenol, medrogestone, medroxyprogesterone acetate, megestrol acetate, mestranol, nomegestrol, nomegestrol acetate, norelgestromin, norethisterone, norethindrone, enanthate, norethynodrel, norgestimate, norgestrel, norgestrienone, polyestradiol phosphate, progesterone, promegestone, quinestrol, synthetic conjugated estrogens a, synthetic conjugated estrogens b, zeranol [29,30].

*Increase* 

Apixaban, benzylthiouracil, boldenone, carbimazole, dabigatran, drostanolone, hermin, methimazole, potassium iodide, potassium perchlorate, propylthiouracil, rivaroxaban, testosterone, testosterone cypionate, testosterone enanthate, testosterone propionate, testosterone undecanoate, tibolone, urokinase [29,30].

Storage and Dosage 

Epoprostenol for injection is a sterile sodium salt formulated for intravenous (IV) administration which is a lyophilized powder for reconstitution with a sterile diluent. When prepared with the existing pH 10.5 diluent, Epoprostenol solution should be administered within a 24-hour period of preparation. It should be maintained within a temperature range of 2°C to 8°C during infusion, which requires the use of a cold pack. Moreover, the cold pack used to keep the temperature of the Epoprostenol solution requires frequent changes. The stability of the Epoprostenol solution could be stored at ambient temperatures by increasing the pH of the diluent from 10.5 (or pH 10.2 to 10.8) to 12.0 (or pH 11.7 to 12.3). The Epoprostenol solution prepared with pH 12.0 diluent can be stored between 2°C to 8°C for a period of up to 8 days and administered up to 24 hours (temperature of up to 35°C) or up to 72 hours (up to 25°C). The change in the new Epoprostenol formulation is limited only to the diluent; the quantity and formulation of the active ingredient, epoprostenol, stands the same [25].

With a short biological half-life [3-6 min], epoprostenol is administered via a central venous catheter by continuous intravenous infusion utilizing an ambulatory infusion pump. During the initiation of treatment, epoprostenol may be administered peripherally. The overall process requires careful preparation and strict hygiene standards [31].

Epoprostenol for injection is infused at a minimum dose of 2 ng/kg/min and increased by 2 ng/kg/min every 15 minutes according to clinical necessity. If a tolerance limit is reached, subsequent increases are not clinically indicated. However, if dose-limiting pharmacologic effects occur, decreasing the infusion rate until epoprostenol is tolerated should be applied. If symptoms of pulmonary hypertension continue or recur, consider increasing the dose. Determine at least 15-minute intervals to evaluate clinical responses while adjusting the infusion by 1 to 2 ng/kg/min increments. During clinical trials, incremental increases in dose occurred at intervals of 24 to 48 hours or longer. After establishing a new chronic infusion rate, the patient should be observed, and heart rate and blood pressure should be monitored in standing and supine positions for several hours to ensure that the new dose is tolerated. The emergence of dose-limiting pharmacological events during chronic infusion can necessitate a decrease in infusion rate; however, the adverse event may resolve without dosage adjustment in some cases. Reduce the dosage steadily by 2 ng/kg/min every 15 minutes or longer until the dose-limiting effects have passed. Avoid abrupt epoprostenol withdrawals or significant decreases in infusion rates. Infusion doses of epoprostenol can only be changed under the supervision of a physician to avoid any life-threatening condition (e.g., unconsciousness, collapse, etc.) [21].

Mechanism of action

Depending on the producer, intravenous epoprostenol is a powder formulation for the solution or injection powder lyophilized. The presentation varies from 0.5 mg/vial, 1.5 mg/vial, 0.5 mg/10 ml, 1.5 mg/5 ml, 1.5 mg/10 ml [32,33]. Epoprostenol is a part of the group of prostacyclin (prostaglandin I2 or PGI2) agents. It is a synthetic form of prostacyclin that occurs naturally in the body and is the major metabolite of arachidonic acid [23,34,35]. It is produced predominantly by endothelial cells and acts on local vasculature and blood cells that adhere to the endothelium and mediate many biological functions [36]. 

Major pharmacological actions of epoprostenol include 1) direct vasodilation of pulmonary and systemic arterial vascular beds and 2) inhibition of platelet aggregation [36,37]. Prostacyclin (PGI2), produced from endothelial cells, activates G protein-coupled receptors on platelets and endothelial cells. This activation triggers adenylate cyclase to produce cyclic AMP, inhibiting further platelet activation and activating protein kinase A [38]. In patients with PAH, epoprostenol triggers immediate vasodilatory action in the pulmonary and systemic circulation, decreases pulmonary vascular resistance (PVR), and improves circulation [23,36].

Safety Profile

Epoprostenol has been proved to be one of the safest treatment protocols for pulmonary hypertension. It is also one of the best treatments to reduce the mortality rate in patients with idiopathic PAH [14]. As mentioned earlier, the treatment approaches for PAH have been developed considering the vasodilator pathways involved in pathophysiology. Therefore major adverse events are associated due to this property of the drugs. Headaches, nausea/vomiting, flushing, myalgias, jaw pain, diarrhea, upper respiratory tract infections are the most frequent adverse events observed. To maintain the safety profile of epoprostenol, dose-dependent adjustments are necessary. In addition, there have been several reconstitution formulations developed due to the limited stability of epoprostenol [38].

To avoid interruptions in drug delivery, the patient should have access to a backup infusion pump and intravenous infusion sets. As intravenous infusion can sometimes be complex, many of the adverse events of epoprostenol are associated with the delivery system and not its mechanism [39].

Epoprostenol has demonstrated safety in pregnant and lactating mothers as well [20]. Unfortunately, there are not many updates about its safety in children with PAH. The data remains limited to few clinical trials and needs further investigation, even in young adults.

There have been few contraindications to the use of epoprostenol. Patients with a history of congestive heart failure and those who develop pulmonary edema are strictly advised not to continue using this drug. It is not recommended to be given along with other parenteral solutions or medications in the same intravenous line. Consider a multi-lumen catheter if other intravenous therapies are routinely administered [40].

Chronic dosage administration of epoprostenol formulations has adverse events such as thrombocytopenia, hypotension, tachycardia, bradycardia, and bleeding [39,41]. During studies, other complications have been observed in a minority of patients which were associated with catheter-related usage, including local infection (18%), pain (11%), and sepsis. Pulmonary embolism and hepatic failure were reported after approval in postmarketing experience, but it was not possible to affirm that the exposure to the drug was the cause [28].

Clinical trials

Current clinical trials have focused on developing new formulations of epoprostenol that may better deliver to the patients. There have been advances such as EPITOME 2 and EPITOME 4 clinical trials [42,43]. These trials proved that the new formulations have a better patient’s convenience-centered approach. 

About 20 clinical trials were completed related to epoprostenol. There are two ongoing clinical trials for epoprostenol (Table 2) [44,45]: 

**Table 2:** 0ngoing clinical trials for Epoprostenol [44,45]

Official Title of the Study	Acute Hemodynamic Comparison of Inhaled Nitric Oxide and Inhaled Epoprostenol in Pulmonary Hypertension	Efficacy of Nebulized PGI2 Versus Nebulized Nitroglycerine for Management of Pulmonary Hypertension After Valve Replacement Surgeries
Identifier number	NCT04231084 [44]	NCT04594629 [45]
Drug	Inhaled Nitric Oxide vs. Inhaled Epoprostenol	Nitroglycerin vs. PGI2 (Epoprostenol)
Phase	N/A	1
Inclusion Criteria	mPAP ≥ 21mmHg, PAWP ≤ 25mmHg, and PVR ≥3 WU; classified as WHO group I-III pulmonary hypertension	35 to 66 years old; elective valve replacement surgery; pulmonary arterial hypertension
Status	Not recruiting yet	Recruiting
Estimated number of subjects	108 participants	120 participants
Country	USA	Egypt

## Conclusions

Epoprostenol has been an essential treatment for pulmonary arterial hypertension (PAH) for the past two decades. It remains a critical treatment option for PAH, with many clinical data supporting its efficacy and tolerability profile. The development of new and more convenient formulations of epoprostenol therapy appears to be reassuring in the management of PAH patients. As its role in patient care keeps evolving, continued studies will determine whether combination therapy is more effective, and new drugs on the horizon may remove the need for continuous infusions. Because the necessity for continuous infusion and the long-standing consequences of these novel treatments are yet unclear, long-term observational studies evaluating various therapies in terms of survival, side effects, quality of life, and costs are deemed necessary. Additional studies that evaluate the effectiveness of epoprostenol compared to new drugs will be needed. 

One of the most significant findings from this study is the necessity to develop new drug formulations. Pulmonary arterial hypertension is not a common disease, but an increasing number of cases have been seen over the last few years. Therefore, more research, including controlled trials, is needed to prove the efficacy of different formulations of epoprostenol. 
